# Operando characterization of cathodic reactions in a liquid-state lithium-oxygen micro-battery by scanning transmission electron microscopy

**DOI:** 10.1038/s41598-018-21503-w

**Published:** 2018-02-16

**Authors:** Pan Liu, Jiuhui Han, Xianwei Guo, Yoshikazu Ito, Chuchu Yang, Shoucong Ning, Takeshi Fujita, Akihiko Hirata, Mingwei Chen

**Affiliations:** 1 School of Materials Science and Engineering, Shanghai Jiao Tong University, Shanghai, 200030 P.R. China; 2 Advanced Institute for Materials Research, Tohoku University, Sendai, 980-8577 Japan; 3 CREST, JST, 4-1-8 Honcho Kawaguchi, Saitama, 332-0012 Japan; 4 Department of Mechanical and Aerospace engineering, Hong Kong University of Science and Technology, Clear Water Bay, Hong Kong SAR; 5 Department of Materials Science and Engineering, Johns Hopkins University, Baltimore, MD 21218 USA

## Abstract

Rechargeable non-aqueous lithium-oxygen batteries with a large theoretical capacity are emerging as a high-energy electrochemical device for sustainable energy strategy. Despite many efforts made to understand the fundamental Li-O_2_ electrochemistry, the kinetic process of cathodic reactions, associated with the formation and decomposition of a solid Li_2_O_2_ phase during charging and discharging, remains debate. Here we report direct visualization of the charge/discharge reactions on a gold cathode in a non-aqueous lithium-oxygen micro-battery using liquid-cell aberration-corrected scanning transmission electron microscopy (STEM) combining with synchronized electrochemical measurements. The real-time and real-space characterization by time-resolved STEM reveals the electrochemical correspondence of discharge/charge overpotentials to the nucleation, growth and decomposition of Li_2_O_2_ at a constant current density. The nano-scale operando observations would enrich our knowledge on the underlying reaction mechanisms of lithium-oxygen batteries during round-trip discharging and charging and shed lights on the strategies in improving the performances of lithium-oxygen batteries by tailoring the cathodic reactions.

## Introduction

Lithium-oxygen (Li-O_2_) batteries have recently attracted enormous research attention as a new generation of high energy storage devices for all electric vehicles and other high-energy-demanded applications because of the high theoretical capacity^1^. However, the practical implementation of Li-O_2_ batteries is facing substantial challenges, particularly, in the development of high performance cathodes and stable electrolytes for high round-trip efficiency, long lifetimes and high rate capability^1–4^. Unlike intercalation reactions in lithium-ion batteries, the electrochemistry of Li-O_2_ batteries is based on a reversible reaction of 2Li + O_2_ ⇔ Li_2_O_2_. The formation and decomposition of the solid Li_2_O_2_ phase during the discharge-recharge cycles *via* oxygen reduction reaction (ORR) and oxygen evolution reaction (OER) are the critical processes which determine the overall battery performances^2–11^. Despite many efforts made to understand the fundamental Li-O_2_ electrochemistry in recent years, the evolution of Li_2_O_2_ on cathodes remains debate with several critical questions which include but are not limited to^9–17^ (1) is the formation of Li_2_O_2_ by a non-Faradaic disproportionation reaction of intermediate LiO_2_ or by a direct electrochemical reduction of O_2_ to Li_2_O_2_? (2) how can the continuous formation of insulating Li_2_O_2_ on the cathode surface keep a constant or near constant discharge potential? (3) does the decomposition of Li_2_O_2_ take place at Li_2_O_2_/electrolyte interfaces or the Li_2_O_2_/electrode interfaces? The investigations of cathodic reactions, associated with the formation and decomposition of Li_2_O_2_, have been conducted extensively by differential electrochemical mass spectrometry^11^, nuclear magnetic resonance^13^, surface enhanced Raman spectroscopy^14^, X-ray diffraction^15^, *ab initio* calculations^16^, *ex situ* transmission electron microscopy (TEM) and scanning electron microscopy (SEM)^17^, and so on. However, the direct correspondence of Li_2_O_2_ nucleation, growth and decomposition to electrochemical discharge/charge in the liquid-state Li-O_2_ system has not been well established owing to the lack of operando characterization with real-space visualization. The rationale of the basic cathodic reaction kinetic process, related to those questions, is still in dispute. Therefore, the direct observations of the nucleation, growth and decomposition of Li_2_O_2_, accompanying with simultaneous electrochemical measurements of discharging/charging response, is highly desired to answer those fundamental questions and thus the reaction kinetics of the Li-O_2_ batteries.

TEM with a high spatial resolution has been demonstrated as a powerful tool for *in situ* characterization of electrochemical and chemical reactions^12,18–20^. In particular, the recently-developed micro-fabricated electrochemical cells (EC) provide a unique advantage for imaging electrochemical reactions while electrochemical measurements can be performed simultaneously. For lithium ion batteries, *in situ* EC TEM and scanning TEM (STEM) have been used to observe lithiation and delithiation dynamics in silicon nanowires and LiFePO_4_ nanoparticle electrodes^21,22^. Although *in situ* TEM has been utilized to study the formation and/or decomposition of Li_2_O_2_ in Li-O_2_ batteries^12,23,24^, electrochemical processes of these studies were performed at a constant voltage and thus cannot provide the information on the electrochemical correspondence of the nucleation, growth and decomposition of Li_2_O_2_ to discharge/charge overpotentials. Moreover, the weak contrast of Li_2_O_2_ under TEM mode, especially interfered by liquid surroundings in EC, limits the spatial resolution for detecting the formation/decomposition of light Li_2_O_2_ under non-equilibrium high-rate charge/discharge conditions. In contrast, the state-of-the-art spherical aberration corrected STEM with enhanced contrast by a high angle annular dark field (HAADF) detector is a promising technique for characterizing basic electrochemical reactions in liquid-state at a high spatial resolution of ~1 nm^19^. In this study we employ *in situ* EC HAADF-STEM to investigate the nucleation, growth and decomposition of Li_2_O_2_ in a basic liquid-state Li-O_2_ system during discharging and charging at a constant current density, together with synchronized electrochemical measurements.

**Figure 1:**
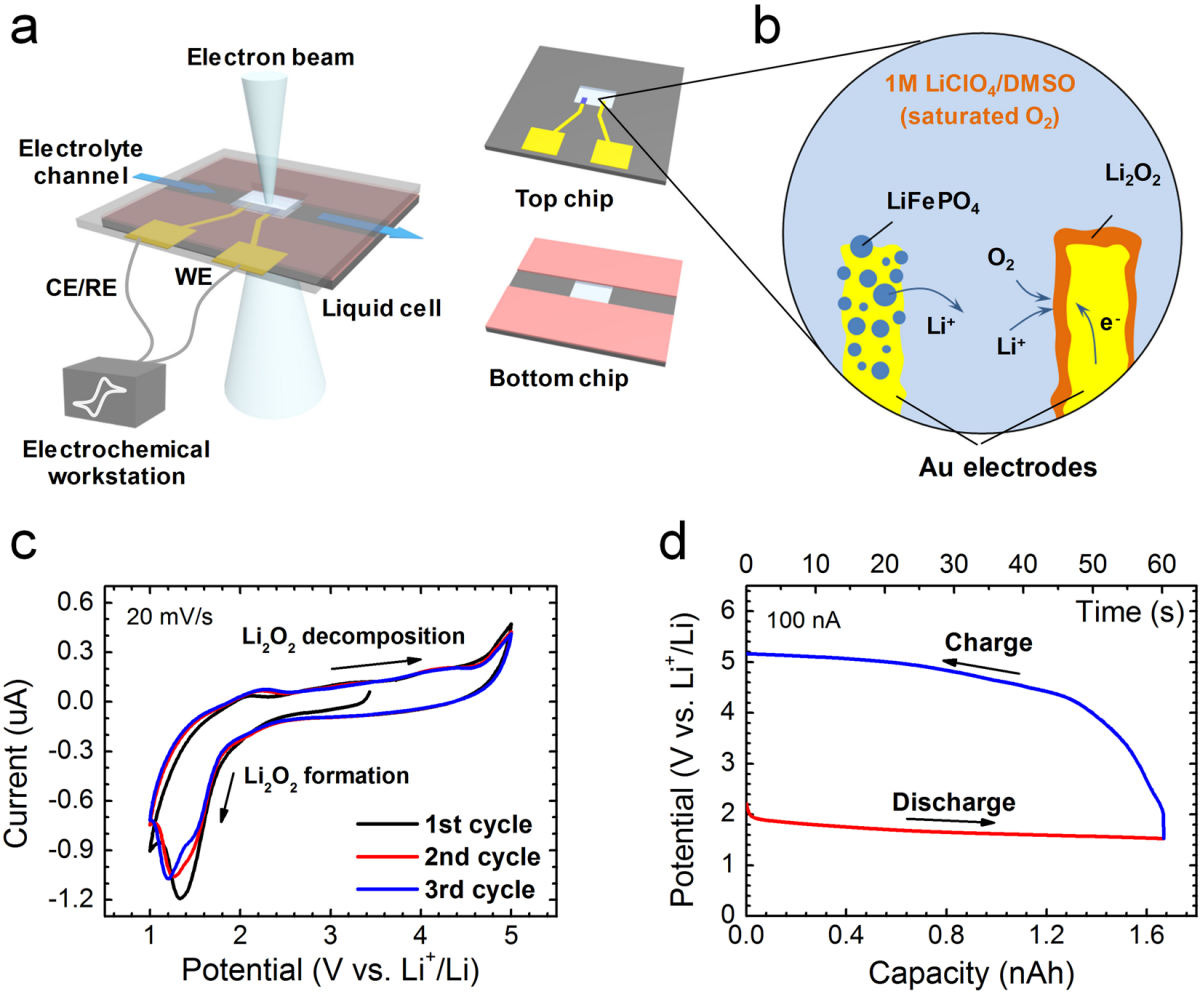
(**a**) Schematic diagram of the Li-O_2_ micro-battery setup for operando STEM characterization of cathodic reactions during charging and discharging. A top microchip is patterned with 120 nm wide Au electrode. (**b**) Schematic reactions in the micro-battery with the formation and decomposition of solid Li_2_O_2_ on the Au cathode during discharge-charge cycles. LiFePO_4_ nanoparticles are loaded on the Au anode as the Li^+^ source. (**c**) Three cyclic voltammetry cycles with voltage range from 1–5 V of the micro-battery measured in TEM liquid holder. (**d**) The synchronized galvanostatic discharge-charge curves of the micro-battery at the current of 100 nA with the cut-off capacity of ~1.67 nAh. The electrolyte is 1 M LiClO_4_ in DMSO.

Figure 1a shows the schematic diagram of the Li-O_2_ micro-battery for *in situ* STEM observations. A micro-channel was sandwiched between two micro-chips with ~30 nm thick electron-transparent Si_3_N_4_ membranes to form a flowing liquid cell. The top chip was patterned with two Au pads as the electrodes for electrochemical measurements. One Au pad loaded with LiFePO_4_ nanoparticles was used as the counter electrode. The formation and decomposition of Li_2_O_2_ during discharge-charge cycles were recorded on the other Au pad which acted as the working electrode (Fig. 1b). 1.0 M LiClO_4_ in dimethylsulfoxide (DMSO) saturated with oxygen was used as the electrolyte and fed into the cell at a low flowing rate of 1 μL/s through an external liquid pump. The flowing electrolyte keeps near constant oxygen and Li ion concentrations during electrochemical measurements and STEM observations at a very low flow velocity (smaller than 0.05 nm/s) of the electrolyte in the vicinity of the Au cathode, calculated by the finite element analysis (Fig. S2). The influence of the flowing electrolyte at the low velocity on the cathodic reaction kinetics is ignorable as verified by invisible mechnical motion of small Li_2_O_2_ nanoparticles. The assembled liquid Li-O_2_ micro-cell was loaded into a TEM holder and connected to an electrochemical workstation. To achieve high-quality images, a Cs-corrected STEM mode with a mass-sensitive HAADF detector was employed for the operando observations. The spatial resolution of the Cs-corrected STEM liquid cell was estimated to be ~1 nm^19,25^. Additionally, HAADF-STEM images were taken at a relatively lower resolution of 512 by 512 pixels and a short scanning time of 5 μs/pixel to avoid electron beam irradiation and sample drift and to capture more frames during observations. Under the imaging condition, the electron dosage is as low as 0.53 e^−^/Å^2^ with low magnification of 50k, which is several orders of magnitude lower than that of the typical high-resolution Cs-corrected STEM (~10^5^ e^−^/Å^2^)^26,27^ (Table S1). The experimental parameters of the low-dose STEM can be found in Supplementary Materials. To test the electrochemical performance of the Li-O_2_ micro-battery inside TEM, the cyclic voltammetry (CV) measurements were performed as shown in Fig. 1c. The CV curves well trace the reversible cathodic reactions and are consistent with those of conventional Li-O_2_ batteries^28,29^, demonstrating that the electrochemical functions of our Li-O_2_ micro-battery are essentially identical to these of conventional coin batteries although their apparent configurations look different. A time-capacity dependent potential evolution curve during galvanostatic discharge and charge at a constant current density of 1.3 μA/cm^2^ was recorded during HAADF-STEM observations (Fig. 1d). In spite of the slightly high charge/discharge overpotentials due to large series resistance and complex configuration of the liquid cell setup^12,30^, the characteristic voltammetric profile of the micro-battery is consistent with those of conventional Au based Li-O_2_ batteries^6,31,32^. Moreover, the galvanostatic charge/discharge profiles do not show obvious changes when the electron beam for STEM imaging turns on and off (Fig. S3). Moreover, we have verified that the electrolyte and reaction product Li_2_O_2_ are stable under the low-dose STEM mode in the experimental time scale (Supplementary Materials). Therefore, the influence of electron beam irradiation on the cathodic reactions during our operando observations should not be observable. Under the applied galvanostatic discharge-charge cycles, the detailed electrochemical reactions on the Au cathodes of the Li-O_2_ micro-battery can be monitored simultaneously by STEM.

**Figure 2:**
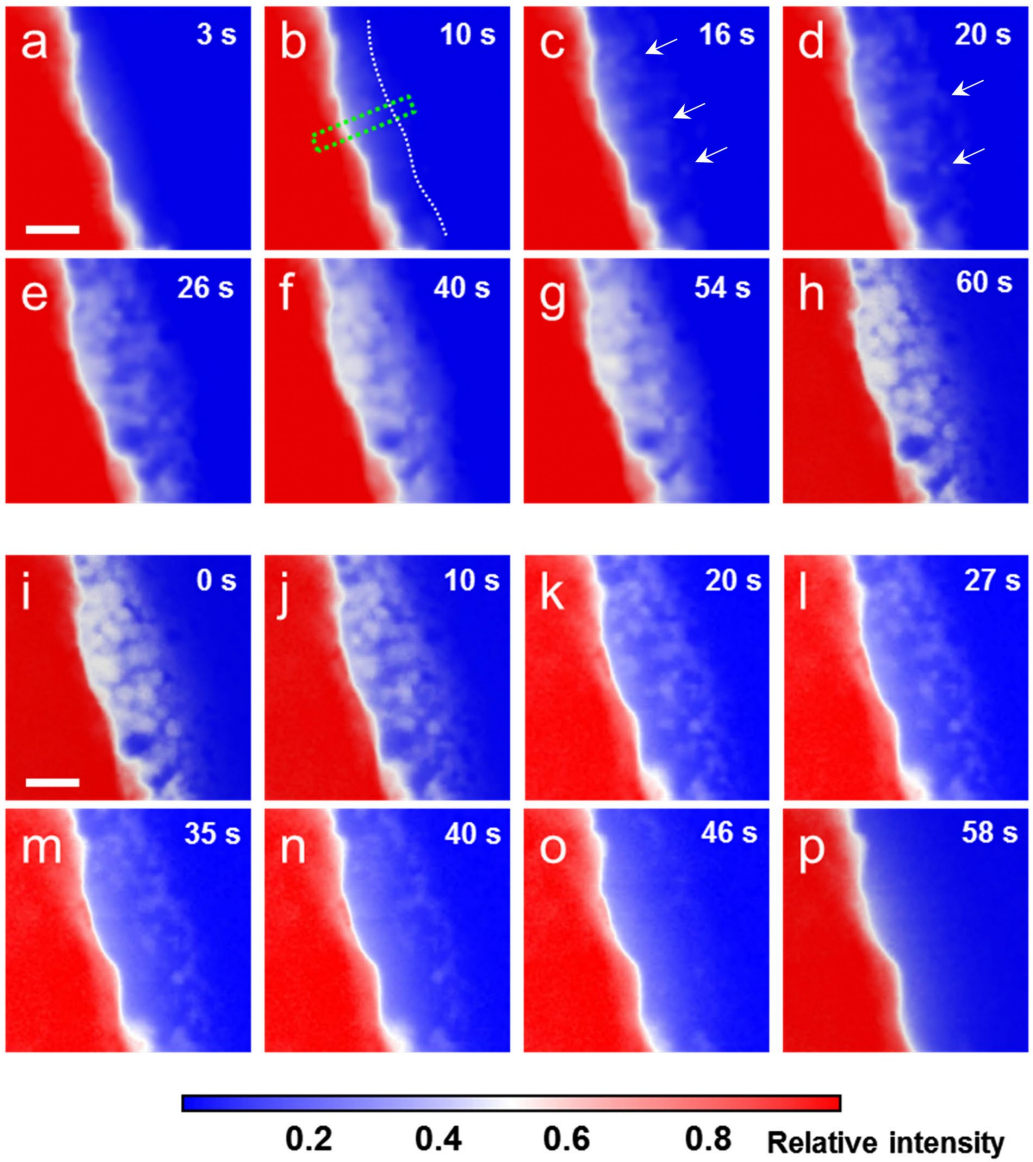
Time sequential HAADF-STEM images during a discharge-charge cycle. The blue-white-red colors are used to enhance the weak Z-contrast of the reaction products of light Li_2_O_2_. The regions with a bright white contrast represent the Li_2_O_2_ phase. (**a**,**b**) A diffuse layer with weak contrast in the vicinity of the charged electrode. (**c**–**h**) Nucleation and growth of Li_2_O_2_ nanoparticles and the formation of a network structural Li_2_O_2_ film during discharging. (**i** and **j**) The preferential dissolution of Li_2_O_2_ at the electrode/Li_2_O_2_ interface. (**k**–**o**) The decomposition of Li_2_O_2_ via the continuous dissolution of Li_2_O_2_ particles at electrolyte/Li_2_O_2_ interfaces during charging. (**p**) A diffuse layer around the gold electrode after the complete dissolution of Li_2_O_2_ particles. (scale bar: 100 nm, E_0_ = 200 keV, magnification: 50 k, dwell time: 5 μs/pixel, image size: 512 × 512 pixels, electron dose: 0.53 e^−^/Å^2^).

The time sequential HAADF-STEM images during discharging are presented in Fig. 2a–h. To enhance the weak contrast of the reaction products of light Li_2_O_2_, color correction with blue, white and red is used. For reference, the original grey HAADF-STEM images are shown in Fig. S15. The regions with a bright white contrast represent the Li_2_O_2_ phase while the very bright rim of the gold electrode in Fig. 2a may be from the edge effect of the electrode because it keeps unchanging with discharging and charging. During the first 10 seconds of the discharging reaction, a diffuse layer with continuous bright contrast emerges, as marked by white dashed line in Fig. 2b. Although discrete Li_2_O_2_ particles cannot be identified from the diffuse layer by HAADF-STEM, correspondingly, the discharge overpotential reaches the discharge plateau with continuous increase of capacity. It is known that the solubility of Li_2_O_2_ in DMSO is very low^7^. The absence of the Li_2_O_2_ phase on the electrode surface and diffuse layer suggests that the discharge reaction is carried out by the one-electron reduction of O_2_ with the formation of lithium superoxide (LiO_2_) intermediates which have high solubility in DMSO. Since the contrast of HAADF images arises from the high-angle electron scattering, the white contrast of the diffuse layer evidences the enhanced electron scattering from the electrolyte in the vicinity of charged electrode. Thus, the enhanced contrast of the diffuse layer most likely originates from the charged or polarized molecules of the electrolyte in electric fields, which may have higher structure ordering by polarized molecule alignment or higher density from reduced inter-molecule distances. This assumption is supported by the fact that the diffuse layer can also be observed around the Au electrodes with a pure DMSO electrolyte without the additives of O_2_ and Li ions (Fig. S5). The thickness of the diffuse layer gradually increases with discharge time and reaches the maximum of about 80–90 nm before the precipitation of solid Li_2_O_2_ phase (Fig. S4). When discharging for about 16 seconds, nanoparticles gradually become visible within the diffuse zone and preferentially start from the topmost surface of the gold electrode. Significantly, the precipitation of isolated nanoparticles in the electrolyte without direct contact with the electrode can also be observed, as marked by arrows in Fig. 2c and d. These observations indicate that the formation of solid Li_2_O_2_ phase is most likely by a non-Faradaic disproportionation reaction lagging behind the one-electron ORR. The earlier precipitation of Li_2_O_2_ in the vicinity of Au electrode surfaces is also in agreement with the reaction mechanism as the formation of Li_2_O_2_ by the disproportionation reaction is expected to be controlled by LiO_2_ diffusion. With continuously discharging, the thickness of the Li_2_O_2_ layer keeps increasing from ~90 nm to ~160 nm mainly by the formation of new nanoparticles from the diffuse layer (Fig. 2d–g). The newly-formed nanoparticles often connect with pre-existed ones and form a network like porous structure. After ~60 seconds, the discharge products forms a ~160 nm thick Li_2_O_2_ layer on the Au cathode, which is composed of loosely interconnected nanoparticles with open pore channels (Fig. 2h).

The formation of Li_2_O_2_ has been suggested to follow a two-step reaction, which includes the one-electron reduction of O_2_ to form the lithium superoxide (LiO_2_) intermediates (Eq. 1) and subsequently either a non-Faradaic disproportionation reaction (Eq. 2) or a second reduction reaction (Eq. 3) to form the final Li_2_O_2_ discharge product^5,14^:

1 $${{\rm{Li}}}^{+}+{{\rm{O}}}_{2}+{{\rm{e}}}^{-}\to {{\rm{LiO}}}_{2}$$

2 $${2{\rm{L}}{\rm{i}}{\rm{O}}}_{2}\to {{\rm{L}}{\rm{i}}}_{2}{{\rm{O}}}_{2}+{{\rm{O}}}_{2}$$

3 $${{\rm{Li}}}^{+}+{{\rm{LiO}}}_{2}+{{\rm{e}}}^{-}\to {{\rm{Li}}}_{2}{{\rm{O}}}_{2}$$

The operando STEM observations provide compelling evidence that the formation of Li_2_O_2_ is through a hybrid electrochemical and chemical process, *i.e*, the one-electron reduction of O_2_ to LiO_2_ by Eq. 1 and followed by the non-Faradaic disproportionation of LiO_2_ with Eq. 2 under the current experimental conditions. The hybrid mechanism allows Li_2_O_2_ nanoparticles to directly precipitate from electrolyte, rather than layer-by-layer growth on the electrode surface. The operando observation is also consistent with the reaction mechanism proposed by Bruce *et al*. The high-donor-number solvent DMSO with a high solubility of LiO_2_ facilitates the formation of Li_2_O_2_ through a solution pathway^28^. It has been reported that the disproportionation of LiO_2_ to Li_2_O_2_ has a half-life of tens of seconds^14^, close to the time scale of the discharge in our *in situ* STEM experiments. Therefore, the direct second electron reduction pathway by charge transfer (Eq. 3) appears unfavorable at a low constant discharging current. As shown in the time sequential HAADF-STEM images, the formation of Li_2_O_2_ is dominated by the nucleation process in the initial stage. The soluble LiO_2_ produced at the electrode surface diffuses into the electrolyte and forms Li_2_O_2_ by the disproportionation reaction (Eq. 2). When the concentration of Li_2_O_2_ exceeds the solubility limit, crystallites precipitate from the electrolyte as the initial Li_2_O_2_ nuclei and gradually grow up during continuous discharging. The smallest Li_2_O_2_ precipitates, which can be identified by the STEM observations, are about 3–4 nm (Fig. S6), which could be the upper bound of the critical size of Li_2_O_2_ precipitates. The formation of the network structure with interconnected Li_2_O_2_ particles may result from heterogeneous nucleation of Li_2_O_2_ nanoparticles during which the pre-existed particles act as the nucleation sites to reduce the nucleation barrier of newly-formed Li_2_O_2_ particles, or attachment growth of the nanoparticles. The open channels in the network structure enable the gold electrode to contact with electrolyte. In this way the LiO_2_ intermediates can be continuously generated from the electrode by ORR at a nearly constant overpotential until the electrode is completely passivated by a solid Li_2_O_2_ film which blocks the contact between the gold electrode and electrolyte (Fig. S13)^33^. The crystal structure and chemistry of the Li_2_O_2_ phase formed during the operando observations are verified by separate selected area electron diffraction (SAED) and electron energy loss spectroscopy (EELS) analysis at a low acceleration voltage of 120 kV (Fig. S12**)**.

**Figure 3:**
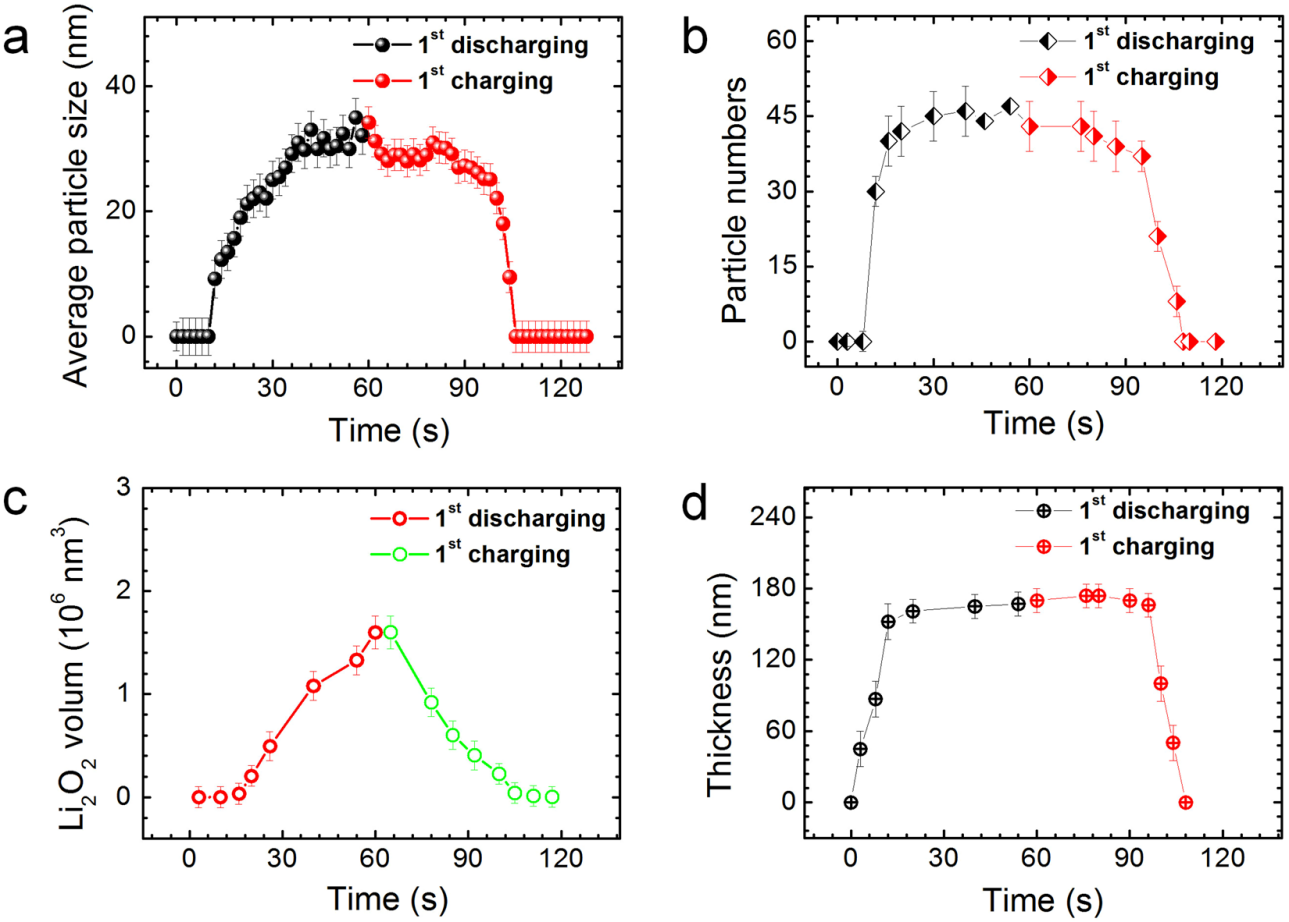
The evolution of Li_2_O_2_ particles during charging and discharging. (**a**) Parabolic relation between average particle size and discharge/charge time. (**b**) Relation between particle number and discharge/charge time. (**c**) Total volume changes of Li_2_O_2_ with discharge/charge time. Note that the volume has a linear relation with time. (**d**) The plot of Li_2_O_2_ layer thickness with discharge/charge time.

The formation kinetics of Li_2_O_2_ was investigated by measuring the evolution of the size and number of Li_2_O_2_ particles during discharging. As shown in Fig. 3a and b, the average particle size *via* discharge time is parabolic while the particle number quickly increases within first 20 seconds and then remains nearly constant, indicating that the formation of solid Li_2_O_2_ phase is via a nucleation-growth process. From the initial discharge plateau to the end of cut-off capacity, the mean particle size increases from ~10 to ~35 nm within ~60 seconds at the average growth rate of 0.4 nm/s. The low growth rate is in line with the low current density used in this study. We also estimated the total volume changes of Li_2_O_2_ with discharge time by assuming the Li_2_O_2_ nanoparticles have an approximate spherical shape (Fig. 3c). Since the liquid layer is very thin (~120 nm), the density of Li_2_O_2_ particles is not very high and the contrast difference between the solid Li_2_O_2_ particles and electrolyte is large, most Li_2_O_2_ nanoparticles can be counted during the *in situ* observations, although a small number of nanoparticles may be missing because of the overlap along the electron beam direction in the early stage of discharging. Interestingly, except the first 10 second corresponding to the formation of the diffuse layer, the Li_2_O_2_ volume has a linear relation with discharge time and thereby capacity, which is consistent with the smooth discharge curve at a constant current density (Fig. 1d). After the initial stage of Li_2_O_2_ formation, the thickness of the Li_2_O_2_ layer keeps nearly unchanged during continuous discharging (Fig. 3d) and the increase of the charge capacity is by the growth of individual Li_2_O_2_ nanoparticles. The nearly constant Li_2_O_2_ layer thickness could be associated with the relatively stable concentration gradient of LiO_2_ around the electrode because the contour of the Li_2_O_2_ layer shows the similarity with the local curvature of the Au electrode. Since Li_2_O_2_ is almost insoluble in the non-aqueous DMSO electrolyte^7^, Li_2_O_2_ precipitates once it forms by disproportionation of LiO_2_. The growth of Li_2_O_2_ particles is more likely controlled by the discharge reaction rate, namely LiO_2_ formation rate and discharge current density, after a stable LiO_2_ concentration gradient is formed in the vicinity of the electrode. Both the linear relation of Li_2_O_2_ volume vs. time and the parabolic curve between Li_2_O_2_ particle size and time are in good agreement with the galvanostatic discharge mode which has a constant LiO_2_ formation rate and thus Li_2_O_2_ formation rate (Li_2_O_2_ volume per unit time) under a near equilibrium condition.

To investigate the decomposition reaction of Li_2_O_2_, galvanostatic charging was conducted after the Li_2_O_2_ formation (Fig. 1d). The corresponding evolution of Li_2_O_2_ particles during charging is shown in Fig. 2i–p. Within the first 10 seconds, noticeable shrinkage of the Li_2_O_2_ nanoparticles takes place at the Au electrode/Li_2_O_2_ interface (Fig. 2i,j), indicating that the preferential decomposition of Li_2_O_2_ takes place from those directly contacting with the electrode. The process is accompanied by a quick increase of the charge potential (Fig. 1d) with the gradual loss of the direct contact between Li_2_O_2_ nanoparticles and the electrode. This observation is consistent with previous *in situ* TEM studies^12,23^ and evidences that the decomposition of Li_2_O_2_ starts from the electrode surface at lower charging potentials. The kinetics of Li_2_O_2_ oxidation is apparently limited by the charge transport between the electrode and indirectly contacted Li_2_O_2_ particles. With further charging, noticeable dissolution of Li_2_O_2_ particles that reside away from the gold electrode (up to 160 nm) can be observed at high charge potentials (Fig. 2k–n). This phenomenon is different from the previous observations that the decomposition of Li_2_O_2_ is carried out by the collapse of Li_2_O_2_ films to keep direct contact between Li_2_O_2_ and electrode for interfacial oxidation operated at constant voltages and high current densities^23^. In our experiment, the charge and discharge are conducted at a constant current density of 1.3 μA/cm^2^, similar to the conventional battery testing, and the charge reaction is under a near electrochemical equilibrium state with a relatively lower reaction rate in comparison with that of ref.^23^. The Li_2_O_2_ oxidation involves charge transfer, *i.e*.

4 $${{\rm{Li}}}_{2}{{\rm{O}}}_{2}\to 2{{\rm{Li}}}^{+}+{{\rm{O}}}_{2}+2{{\rm{e}}}^{-},$$

during which considerable electron transport in the porous Li_2_O_2_ film is required for the decomposition of solid Li_2_O_2_ via OER during charging. It is known that bulk Li_2_O_2_ is insulator and electrons cannot tunnel into Li_2_O_2_ deeper than ∼5–10 nm^34^. However, if the Li_2_O_2_ phase formed by ORR during discharging is an insulator, it is difficult to explain the operando observations of continuous oxidation of Li_2_O_2_ nanoparticles far away from the electrode. Thus, one possible explanation is that the Li_2_O_2_ nanoparticles formed by the solution precipitation may be electrically conductive, or at least Li_2_O_2_ particle surfaces are metallic as theoretically predicted by Siegel *et al*.^16,35^. As the reaction product layer consists of inter-connected Li_2_O_2_ particles, the particle surfaces and the interfaces could provide conductive paths for the decomposition of Li_2_O_2_ at Li_2_O_2_/electrolyte interfaces. However, after partial dissolution, many Li_2_O_2_ particles become isolated with the loss of the surface conductive pathway (Fig. 2k). Significantly, the operando observation reveals that the subsequent charging proceeds *via* continuous dissolution of the isolated Li_2_O_2_ particles while the charge potential smoothly increases (Fig. 1d), showing similar behavior in the final charging stage of conventional Li-O_2_ batteries. Therefore, the isolated residual Li_2_O_2_ nanoparticles are not “flotsam” but contribute to the charge recovery by OER. Except the hypothesis of the conductive Li_2_O_2_, the operando observations can also be interpreted by a hybrid chemical and electrochemical process, *i.e*, the dissolution of Li_2_O_2_ by the reverse non-Faradaic disproportionation reaction of Eq. 2: Li_2_O_2_ + O_2_ → 2LiO_2_, and OER by the one-electron oxidation of LiO_2_ by reverse Eq. 1: LiO_2_ →Li^+^ + O_2_ + e^−^. In this case, the charging kinetics is controlled by the non-Faradaic dissolution of Li_2_O_2_, which depends on the solubility of both LiO_2_ and oxygen in the electrolyte. Although recent observations of a redox mediator assisted Li_2_O_2_ decomposition provides an additional clue on the existence of non-Faradaic disproportionation reaction^36^, confirmation of this hypothesis still needs more electrochemical and theoretical evidences. After charging for 58 seconds, all the Li_2_O_2_ particles completely disappear but leave behind a diffuse layer around the Au electrode (Fig. 2p), similar to the initial stage of discharging. The much stronger intensity of the diffuse layer in Fig. 2p than that in Fig. 2a suggests that the formation and distribution of the diffuse layer are associated with the applied potentials of the electrode. It is worth noting that after Li_2_O_2_ nanoparticles completely dissolve at 58 seconds (Fig. 2p) the charging continues until 60 seconds (Fig. 1d). The short charging time gap could be caused by that the remained Li_2_O_2_ nanoparticles are too small to be seen from the low magnification STEM images, the oxidation process of Li_2_O_2_ takes place at other location out of the observation area, or the dissolved LiO_2_ in the electrolyte contributes to the continuous charging by the one-electron oxidation reaction. The measurements of Li_2_O_2_ evolution during charging reveal that the structural evolution of these particles is almost the inverse process of Li_2_O_2_ formation in discharging with the nearly symmetric plots shown in Fig. 3, although the reaction kinetics and reaction pathways for discharging and charging are very different. On the basis of the operando observations (Movie S1), the ORR and OER processes under round-trip discharging/charging cycles at a constant current density are schematically illustrated in Fig. S16.

We noticed that Luo and his co-workers very recently reported *in situ* observations of cathodic reactions in a solid-state Li-O_2_ battery using environmental transmission electron microscopy^24^. This work reveals new insights into oxygen reduction and oxygen evolution reactions in the solid-state Li-O_2_ system. However, it is fundamentally different from our work on the cathodic reactions in the liquid-state Li-O_2_ battery. Electrochemically, the utilization of different electrolytes (liquid versus solid) has rendered them as essentially different systems in the following aspects: (1) the chemistry of the discharge products is different between the two observations. In the study of Luo *et al*., a mixture of Li_2_O_2_ and Li_2_O is formed as the discharge product while in our liquid-state Li-O_2_ system the final products of discharge are only solid Li_2_O_2_. (2) The morphology of discharge products is different. Luo *et al*. reported the formation of hollow spherical particles with Li_2_O outer-shell and Li_2_O_2_ inner-shell surfaces. In the liquid system, the discharge products of Li_2_O_2_ are solid spherical particles. (3) More importantly, the reaction pathways are different. For the solid-state Li-O_2_ battery, the ORR on CNTs initially produces solid LiO_2_ covered by Li_2_O. The LiO_2_ subsequently disproportionates into Li_2_O_2_ and O_2_ through a solid-state transformation reaction. While, in our Li-O_2_ micro-battery with the liquid electrolyte, the LiO_2_ firstly produced is soluble in the electrolyte (in the forms of solvated Li^+^ and O_2_^−^), and upon the disproportionation of LiO_2_, Li_2_O_2_ is formed by nucleation and growth in the liquid electrolyte. In addition to the above differences, there are several unique advantages of our liquid-state Li-O_2_ experimental setup: (1) our liquid-cell Li-O_2_ micro-battery directly accommodates a liquid-state aprotic electrolyte of 1.0 M LiClO_4_ in DMSO with saturated oxygen and is operated under ambient pressure. This environment is almost identical to that of a practical non-aqueous Li-O_2_ battery. In contrast, the environmental TEM used by Luo *et al*. employed a constant O_2_ pressure of 0.1 mbar. Although it was claimed to be oxygen-rich environment, the pressure is far below the atmospheric pressure of a practical solid-state Li-O_2_ battery. (2) In our operando experiments, the charging and discharging were conducted at galvanostatic mode with a constant current density of 1.3 μA/cm^2^, similar to the coin battery testing. In contrast, *in situ* study in the solid-state cells by Luo *et al*. was discharged or charged by applying constant potential biasing and therefore the electrode reactions occurred at a far-from-equilibrium state. Therefore, our experimental setup better resembles the condition of practical batteries and hence the observed phenomena in our operando study would be more translatable for normal real-world batteries. Therefore, these two works investigate quite different systems and, we believe, both are of great scientific significance and would contribute to better understandings of the Li-O_2_ electrochemistry. In particular, the comparison between these two studies would provide insights to how the state of electrolytes affects the fundamental electrochemical reactions and the operation of Li-O_2_ batteries.

In summary, we have developed a liquid-state Li-O_2_ micro-battery for operando characterization of fundamental cathodic reactions under aberration-corrected STEM (STEM) synchronized with electrochemical measurements. The direct observations of Li_2_O_2_ nucleation, growth and decomposition at known discharge/charge overpotentials shine lights on the underlying mechanisms of cathodic reactions at a constant current density. The formation of porous Li_2_O_2_ layer through solution precipitation by the non-Faradaic disproportionation reaction of intermediate LiO_2_ is critical to keep a constant or near constant discharge potential for a large capacity. The dissolution of resultant Li_2_O_2_ nanoparticles during charging is mainly contributed by the Li_2_O_2_/electrode interfacial oxidation at the early stage of the charging reaction. We propose that the hybrid chemical and electrochemical process would be responsible for the oxidation of Li_2_O_2_ particles away from the electrode surfaces. Inspired by the operando observations, we have developed a full performance nanoporous graphene based Li-O_2_ battery by utilizing stable high-donor-number organic solvent DMSO as the electrolyte to enhance the solution mechanism of Li_2_O_2_ formation, the large surface area of nanoporous graphene for multiple heterogeneous nucleation/growth of small and porous Li_2_O_2_ nanoparticles, and an oxidation redox mediator to effectively reduce the charge overpotential by improving the charge transfer between insulating Li_2_O_2_ and electrode^37^.

## Electronic supplementary material

Supplementary Materials

Supporting Movie S1
