# Supplementary material for: Operando characterization of cathodic reactions in a liquid-state lithium-oxygen micro-battery by scanning transmission electron microscopy
Source: Sci Rep. 2018 Feb 16;8:3134. doi: 10.1038/s41598-018-21503-w (PMC5816613; doi:10.1038/s41598-018-21503-w)
Supplement: Supplementary file 1 — Supplementary Materials [file 41598_2018_21503_MOESM1_ESM.docx]

**Supplementary Materials for**

**Operando characterization of cathodic reactions in a liquid-state lithium-oxygen micro-battery by scanning transmission electron microscopy**

Pan Liu**^a, b, c, 1^**, Jiuhui Han**^b, 1^**, Xianwei Guo**^b, c, 1^**, Yoshikazu Ito**^b, c^**, Chuchu Yang**^b^**, Shoucong Ning**^b, d^**, Takeshi Fujita**^b^**, Akihiko Hirata**^b, c^**, Mingwei Chen**^a, b, c, e^***

^a^School of Materials Science and Engineering, Shanghai Jiao Tong University, Shanghai 200030, PR China.

^b^Advanced Institute for Materials Research, Tohoku University, Sendai 980-8577, Japan

^c^CREST, JST, 4-1-8 Honcho Kawaguchi, Saitama 332-0012, Japan

^d^Department of mechanical and aerospace engineering, Hong Kong University of Science and Technology, Hong Kong SAR

^e^Department of Materials Science and Engineering, Johns Hopkins University, Baltimore, MD 21218, USA

^1^ These authors contributed equally to this work.

Corresponding author: [mwchen@jhu.edu](mailto:mwchen@jhu.edu) (M.W. Chen).

**Materials and Methods**

**The setup of lithium-oxygen micro-battery.**

The micro-battery was built based on a liquid cell from Hummingbird Scientific. A 30-50 nm thick low stress silicon nitride film was evaporated on the silicon wafer (200 µm, p-doped) as a membrane of the STEM viewing window. The dimensions of the windows are 50 µm × 200 µm. Two 120 nm-thick gold electrodes were deposited on the top chip of the liquid cell with a face-to-face distance of about 30 µm. The bottom and top chips were separated by the Au electrode spacer. An oxygen saturated 1M LiClO4 DMSO solution was loaded into the reservoirs with a syringe as the electrolyte of the Li-O_2_ micro-battery. The electrolyte was forced to flow through the viewing window by an external pump. The dimensions of top chip and bottom chip are 3 mm × 4 mm and 3 mm × 3 mm respectively, and the working and counter electrodes on the biasing cell were extended to contact with the liquid holder. The electrochemical process was controlled and measured by an electrochemical workstation (Model 660D series made by CH Instruments) connected with the TEM holder. Cyclic voltammetry and galvanostatic charge/discharge measurements were performed during operando observations.

To minimize the influence of flowing electrolyte on the cathodic reaction kinetics, the liquid cell was designed to have three parallel flow channels with different widths as shown in **Figure S1.** Because the width and height of the side channels (150 μm, >400 μm) are much larger than that of the micro-channel (50 μm, ~120 nm), most of electrolyte flows along two wide side channels, which do not involve into electrode reactions but play the role in keeping the electrolyte in the cell to has a near constant oxygen and Li ion concentrations. Only a small amount of liquid slowly flows through the narrow micro-channel for electrode reactions. Moreover, in our study, we applied a low flowing rate of 1 μL/s through external pump driver, which is expected to keep a near constant oxygen concentration in the electrolyte but not affect the cathodic reaction kinetics obviously. The fluid field inside the liquid channel in the sandwiched two electronic chips is estimated by employing the finite element (FE) analysis based on the real configuration of our MEMS based liquid cell (**Figure S1**). The calculated fluid field suggests that only creep flow takes place in the micro-channel due to the very low Reynolds (<<1) which is determined by the fluid cross section radius ($l$), viscosity ($\mu$), and density ($\rho$) ($Re=\rho vl/\mu$). We used Comsol Mutiphysics to conduct the creep flow calculation in the micro-channel with the dimensions of 0.12 × 50 × 3000 μm. The density of DMSO is 1.104 g/cm^3^, and dynamic viscosity is 1.996 cp (1cp=0.001 Pa·s). According to our setup, the inlet pressure is about 20 pa and the outlet pressure is close to 0 pa. Since the viscous effect is much stronger than inertia, the actual flow velocity in the vicinity of the electrode (the observation area of cathodic reactions) is less than 0.05 nm/s, two orders of magnitude smaller than the flow velocity in the center of liquid channel ~4 nm/s (**Figure S2**). Thus, the electrolyte around the edge of electrode can be considered in a static state in the period (a few minutes) of our operando observations.

**Water content in the electrolyte and experimental atmosphere.**

To avoid water contamination, a dry electrolyte with desiccant was stored in a glove box under Ar atmosphere. The water concentration (~12 ppm) in the dry electrolyte was measured by Karl-Fischer titration. The assembly of Li-O_2_ micro-battery in the liquid cell was also conducted in an Ar-filled glove box which has water content lower than ~0.5 ppm and O_2_ content below 4 ppm. Thus, the low water content should not affect the ORR process of our micro-battery as evidenced with the formation of Li_2_O_2_ products which have been confirmed by SAED and TEM-EELS. Moreover, we intentionally exposed the discharged liquid cell into air about 30 min and then checked the changes of the Li_2_O_2_ reaction products. We found that the Li_2_O_2_ phase disappears and, instead, Li_2_CO_3_ phase forms. The out cell reaction further confirms that the original reaction products in our Li-O_2_ micro-battery is Li_2_O_2_. In conclusion, our micro-battery works in a very clean environment and obvious side reactions cannot be detected.

**STEM Characterizations.**

JEM-2100F electron microscope (JEOL) equipped with two aberration correctors (CEOS GmbH) for probe-forming and imaging lens systems was employed for operando STEM characterization with the probe convergence angle of about 29 mrad. A HAADF detector with an inner angle greater than 100 mrad was used for HAADF-STEM. The collect angle of the HAADF detector was set between 100 and 267 mrad. The scanning rate of electron-probe was is 5 μs per pixel and the image resolution is 512 by 512 pixels. Real-time videos of the electrochemical experiments were recorded with ~0.76 frame per second. For better analysis of the experimental results, maximum entropy estimation method (MEEM) was employed to extract quantitative information from the HAADF-STEM images, which gives good signal-to-noise data within a reasonable processing time. The chemical analyses were conducted by X-ray energy-dispersive spectroscopy (JED-2300T, JEOL) and electron energy loss spectroscopy (Gatan GIF system).

**Low-dose operando STEM experiments.**

In our study, we used a viewing mode with a very short dwell time of 5 μs/pixel to record the charge-discharge process, instead of the conventional acquisition mode. The viewing mode is expected to minimize the possible influence of electron beam irradiation. In conventional high resolution Cs-corrected STEM, 38.2 μs/pixel dwell time, 50 pA gun current and 0.11 Å^2^ pixel area are usually employed to achieve high-resolution STEM imaging. Each frame was recorded with 512 × 512 pixels over 10 s, with a field of view ~ 17 × 17 nm^2^, The electron radiation dose is up to about 1.1×10^5^ e^-^/Å^2^ (*Journal of Electron Microscopy 59(2): 103–112 (2010)*). While in our experiments, the combination of the short dwell time 5 μs/pixel and a large pixel area of 1000-8100 Å^2^ at low magnifications (30-80k) gives rise to a very low electron radiation dose of 0.19-1.56 e^-^/Å^2^, which is several orders of magnitude lower than that of the typical aberration-corrected STEM (~10^5^ e^-^/Å^2^) (*Vogt, T., Dahmen, W. & Binev, P. (2012) Modeling Nanoscale Imaging in Electron Microscopy. Springer, Ottawa, Canada.*). Thus, the viewing mode used in our study can effectively reduce the influence of electron beam at the large extent. In comparison with the electron doses and dose rates in recent works (3.37 e^-^/Å^2^s for *ACS Nano, 2012, 6, 8599;* 3.4-13.7 e^-^/Å^2^ frame for *Nano Lett, 2014, 14, 1293;* 30 e^-^/Å^2^s for *Nano Lett, 2015, 15, 2711;*0.25-0.5 e^-^/Å^2^ for *ACS Nano, 2015, 9, 4379;* 150-630 e^-^/Å^2^ for *Nano Lett., 2013, 13, 2964*), the dosage in our study listed in **Table S1** is relative lower and only about 0.19-1.56 e^-^/Å^2^.

**Table S1**. The electron dosages under different dwell times and magnifications and the red ones are the experimental conditions of our study.

| Dwell time (μs/pixel) | Gun current (PA) | Pixel area  (Å^2^) | Magnification | Dosage  (e^-^/Å^2^) |
| --- | --- | --- | --- | --- |
| 38.2 | 50 | 0.11 | 8M | 1.1×10^5^ |
| 5 | 50 | 8100 | 30k | 0.19 |
| 15 | 50 | 8100 | 30k | 0.57 |
| 5 | 50 | 2916 | 50k | 0.53 |
| 5 | 50 | 1000 | 80k | 1.56 |
| 15 | 50 | 1000 | 80k | 4.68 |
| 20 | 50 | 529 | 120k | 11.8 |
| 25 | 50 | 529 | 120k | 14.7 |
| 20 | 50 | 324 | 150k | 19.2 |
| 25 | 50 | 196 | 200k | 39.8 |

**The influence of electron beam on the stability of products Li_2_O_2_.**

To clarify the influence of electron beam irradiation on the cathodic reactions, we established an *ex situ* experimental setup to determine the critical electron dose that leads to Li_2_O_2_ damage. During the observations (both of TEM and STEM modes), the sample Li_2_O_2_ keeps unchanged by using electron dosage of ~11.8 e^-^/Å^2^ even for long time far beyond the time scale of our operando observations. With the increase of the radiation dose by increasing magnification, dwell time and gun current, the volume shrinkage of Li_2_O_2_ particles takes place, implying possible phase transition. In this way, the critical electron dose for the product damage is estimated to ~14.7 e^-^/Å^2^. The TEM-EELS spectra verify that a new Li_2_O phase is produced at a high electron dosage of 1190 e^-^/Å^2^ as the result of electron beam damage of Li_2_O_2_ (**Figure S7** and **S8**). Therefore, the reaction product Li_2_O_2_ appears safe during our operando observations with a very low electron dosage of 0.19-1.56 e^-^/Å^2^. Actually, the fact that the reaction products can be completely dissolved during charging also indicates the influence of electron beam irradiation is minor during the operando observations because the stable Li_2_O phase cannot be oxidized at the applied voltage of 5V.

**Effect of electron beam irradiation on the stability of electrolyte.**

We also investigated the effect of electron beam irradiation on the stability of the electrolyte. The electrolyte in the liquid cell is exposed to the electron beam to see if there is the beam induced electrolyte decomposition. As shown in the **Figure S9**, the electron doses were controlled by magnifications. The **Table S2** shows the relationship between decomposition time of the electrolyte and beam irradiation area at different magnifications. For the same dwell time (10 μs/pixel) and beam current (50 pA), the stability of the electrolyte shows strong dependence on magnification (pixel area) and irradiation time. Within 3 min, no obvious electrolyte decomposition can be seen even under a high electron dose ~9.6 e^-^/Å^2^ (magnification of 150k) (**Figure S10**). It takes more than 1000 sec for the electrolyte decomposition under the magnification of 30k (radiation dosage of 0.38 e^-^/Å^2^, close to our operando observations). Therefore, the electrolyte is stable under our operando STEM observation conditions.

**Table S2.** The required periods for electrolyte decomposition at different magnifications.

| Magnification | 200k | 150k | 100k | 80k | 50k | 30k |
| --- | --- | --- | --- | --- | --- | --- |
| Area (nm^2^) | 4.6E+05 | 8.2E+05 | 1.8E+06 | 2.9E+06 | 7.3E+06 | 2.0E+07 |
| Time (s) | 45 | 180 | 210 | 240 | 390 | > 1000 |
| Electron dose (e^-^/Å^2^) | 15.9 | 9.6 | 4.27 | 3.12 | 1.07 | 0.38 |

**Effect of applied voltage on the stability of electrolyte.**

We measured the critical voltage which can trigger the electrolyte decomposition in our Li-O_2_ micro-battery. The CV curve at the wide potential range of 0-6V was collected at a sweep rate of 20 mV/s. In our setup, the critical decomposition potential of the electrolyte is measured to be about 5.2 V, above which rapid increase of electrode current takes place (**Figure S11**). Therefore, the electrolyte does not experience obvious decomposition during our operando observations with the maximum applied voltage of 5.0 V in the liquid cell during charging. The relatively high critical voltage for electrolyte decomposition obtained in the micro-battery could be caused by the unique microscopic configuration and high internal resistance of liquid cells but hard to be quantified due to the complex of the experimental setup (*Nano Lett., 2011, 11, 4188*). It is worth noting that the charge voltages of Li-O_2_ micro-batteries are usually higher than that of conventional coin cells. For example, a very high charge voltage of ~8V has been reported by a Li-O_2_ micro-battery experiment (*Nano Lett., 2013, 13, 2209*). Actually, such high voltage cannot be true since it is far beyond the decomposition voltage of Li_2_O_2_. For our micro-battery system, the charging voltage of 5.0 V is about 1.0 V higher than that (about 4.0V) of coin batteries with a gold electrode and the same electrolyte (Guo, et al., *Scientific Reports* *2016, 6, 33466*). Thus, the real voltage applied to the gold electrode and surrounding electrolyte in the micro-battery is probably only about 4.0V.

**Reaction production characterization**.

The reaction products after discharging were characterized by selected area electron diffraction (SAED) and electron energy loss spectroscopy (EELS) as shown in **Figure S12.** The crystal structure of the reaction products can be well indexed to be a hexagonal nanocrystalline Li_2_O_2_ phase (*P6_3_/mmc*). The chemical information of the reaction products is determined by EELS by comparing the core-loss spectra with the standard Li_2_O_2_, Li_2_O and Li_2_CO_3_ phases. The distinctive features of Li K-edges suggest that the reaction products are Li_2_O_2_ phase based on the good match in two characteristic peaks (at 60.8 and 65.3 eV) of the EELS spectra between the standard Li_2_O_2_ sample and the reaction products (**Figure S12**). Thus, based on both SAED and EELS results, we can unambiguously conclude that the reaction products are Li_2_O_2_. This conclusion is also supported by the fact that the reaction products can be fully decomposed during charging as shown in **Figure 2**, while the side reaction product Li_2_CO_3_ cannot be fully dissolved during charging.

**Simulations of electric fields around a charged Au electrode.**

A direct current analysis is conducted based on Ansys Maxwell platform, which extends the finite element method (FEM) to the solution of Maxwell equation (**Figure S14**). 100000 triangles are meshed and much intense meshes are generated around the tip of electrodes to ensure precision. The solution type is set as DC conduction, and 2.5v and 0.0v excitation are assigned to the Au electrodes, respectively. The conductivity of the DMSO electrolyte is set as 4.0 siemens/m.

**HAADF STEM simulations.**

To investigate whether the Li_2_O_2_ particles are perfectly overlapped or not, we quantitatively estimate the evolution of intensity profile by HADDF STEM image simulations. The image simulations are performed on the models with the HREM image simulation software using the microscope parameters in **Table S3**. The particle-electrolyte models in the simulations are constructed by positioning a 10 nm hexagonal crystalline Li_2_O_2_ particle in the middle of the electrolyte. Two overlapped Li_2_O_2_ particles are also constructed in the inner of electrolyte for comparison (**Figure S17**). The defocus is set to 10 nm below the particle's topmost atoms and the image pixel size is 1 × 1 nm. The electrolyte is constructed by filling a 30 × 30 × 45 nm box with randomly positioned carbon/oxygen/hydrogen atoms with the same overall density (1.104 g/cm^3^) and stoichiometry as 1.0 M LiClO_4_ in dimethylsulfoxide (DMSO). To avoid channeling effects and to simulate the random orientation of the particles seen in the image, the particle is rotated by 10 degrees along the c-axis, and the contrast is averaged. To avoid surface interaction effects, the DMSO atoms close to the particle atoms are removed. The probe size is set to a large value of 6 nm, to emphasize the general contrast features instead of atomic resolution image contrast. The simulated images show that there is a clear enhanced contrast (~2.3 times) by intensity line scan in the dark field images of the Li_2_O_2_ nanoparticle with overlapped arrangement (see **Figure S17**). Therefore, we can distinguish the 3-D space arrangement from the image simulations, and estimate the particle thickness (or particle numbers) from the intensity profiles.


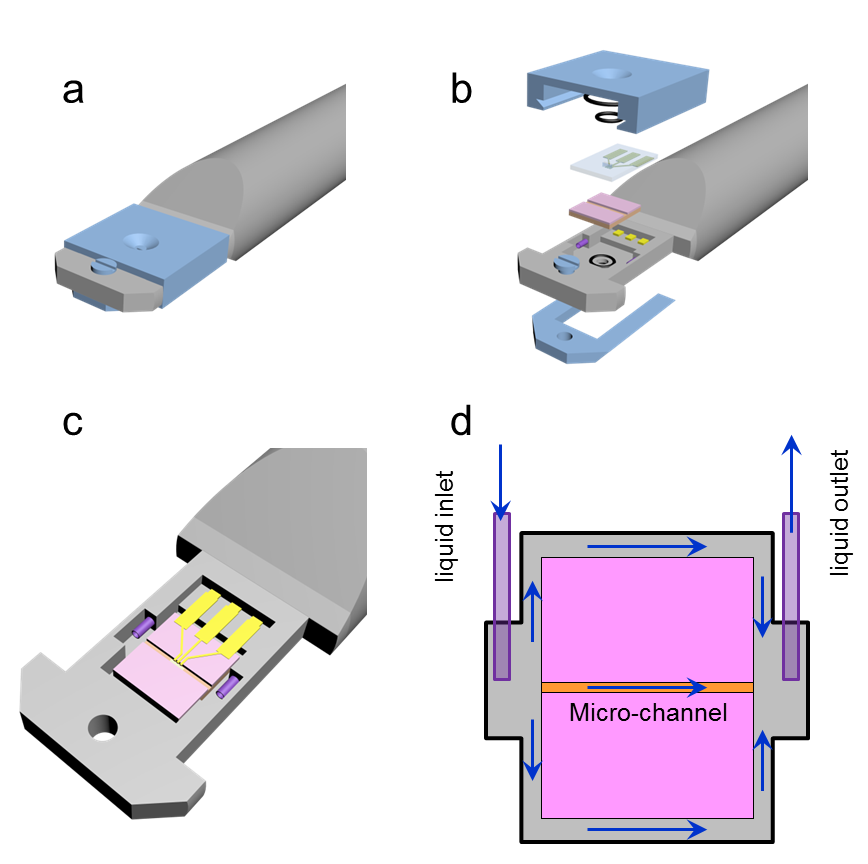


**Figure S1**. Three dimensional configuration of the Li-O_2_ micro-cell. The liquid flowing directions are denoted by blue arrows. Most of liquid flows through two wide side channels and only a small amount of electrolyte slowly passes through the micro-channel.


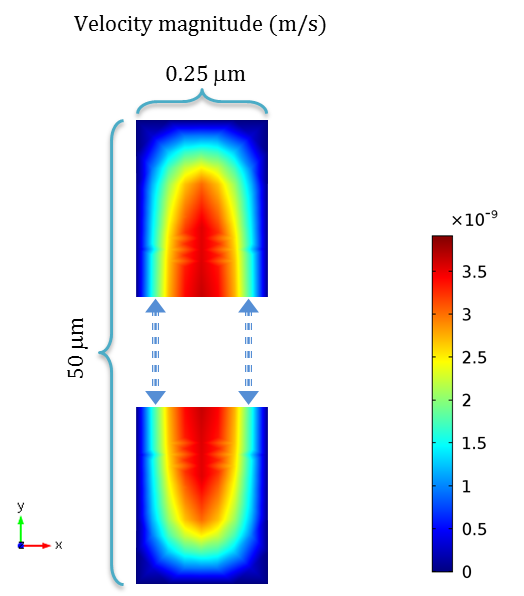


**Figure S2**. The fluid filed within the micro-channel calculated by finite element method based on the real configuration of the liquid cell. The actual flow velocity in the vicinity of the micro-channel wall (Au electrode) is about two orders of magnitude lower than that in the channel center.

**Figure S3**. The galvanostatic charge/discharge profiles of the Li-O_2_ micro-battery recorded during ON and OFF of the electron beam for STEM images. Obvious changes of the profiles cannot be seen when the electron beam for STEM imaging turns on or off.


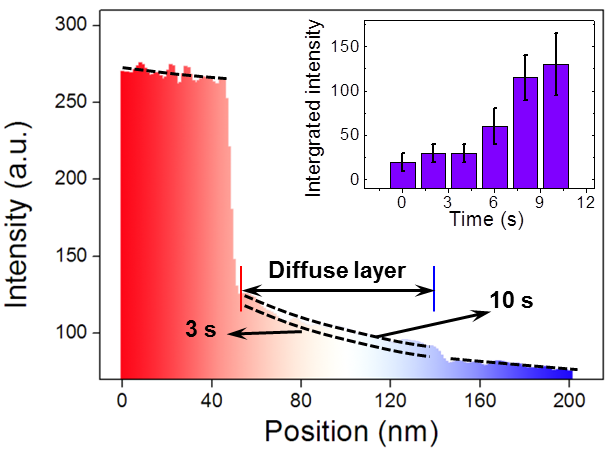


**Figure S4**. The thickness and intensity changes of the diffusion layer with time in the early stage of discharge. The maximum thickness shown in **Figure 2b** is measured to ~80-90 nm from the intensity profile.


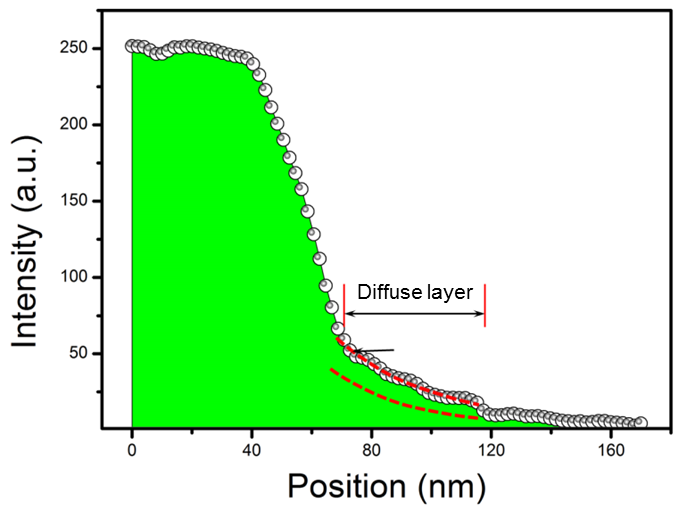


**Figure S5**. The intensity profile of a diffuse layer around the gold electrodes in pure DMSO electrolyte without the additives of O_2_ and Li ions.

**Figure S6**. Measurements of the critical size of Li_2_O_2_ precipitates. (a) STEM image taken from 12 s discharged micro-battery. The smallest Li_2_O_2_ precipitates have a diameter of ~ 3-4 nm, as indicated in the insertion by intensity line scan. This snapshot is extracted from in situ video at the discharge time of ~ 12 s. (b) Statistic size distribution of Li_2_O_2_ precipitates in the nucleation stage. The particle size ranges from 3.6 to 13.7 nm and the upper bound of the critical size of Li_2_O_2_ precipitates is ~3-4 nm.


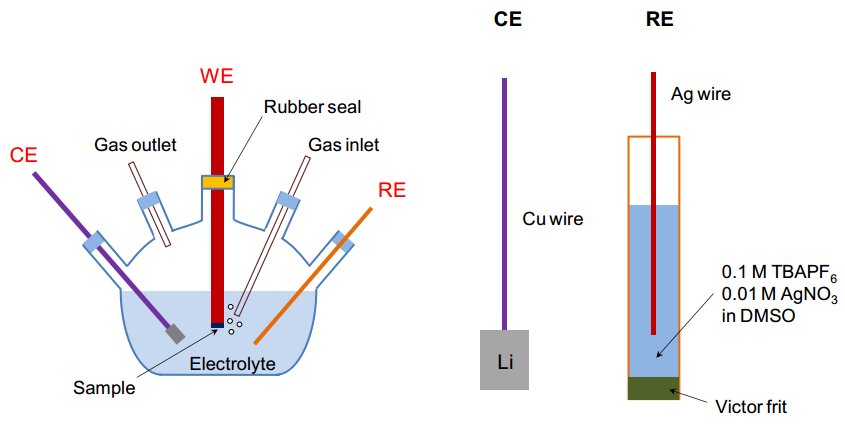


**Figure S7**. Schematic diagram of the home-made three-electrode *ex situ* electrochemical system, which was used to prepare the Li_2_O_2_ samples. The samples were transferred into the vacuum sample transferring holder inside the glove box to measure the critical electron dose of Li_2_O_2_ products damage.


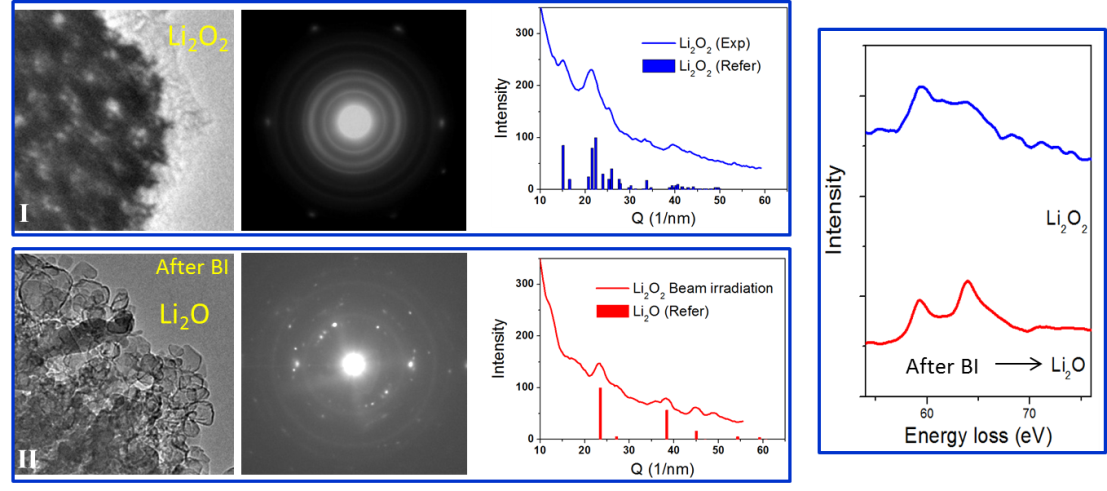


**Figure S8**. SAED patterns and TEM-EELS spectra verify the phase transition from Li_2_O_2_ to a stable Li_2_O phase. The SAED pattern of Li_2_O_2_ was acquired with a electron dose ~ 10 e^-^/Å^2^, lower than the critical electron dose ~14.7 e^-^/Å^2^ for Li_2_O_2_ damage. The electron dose used for beam irradiation is about 1190 e^-^/Å^2^.


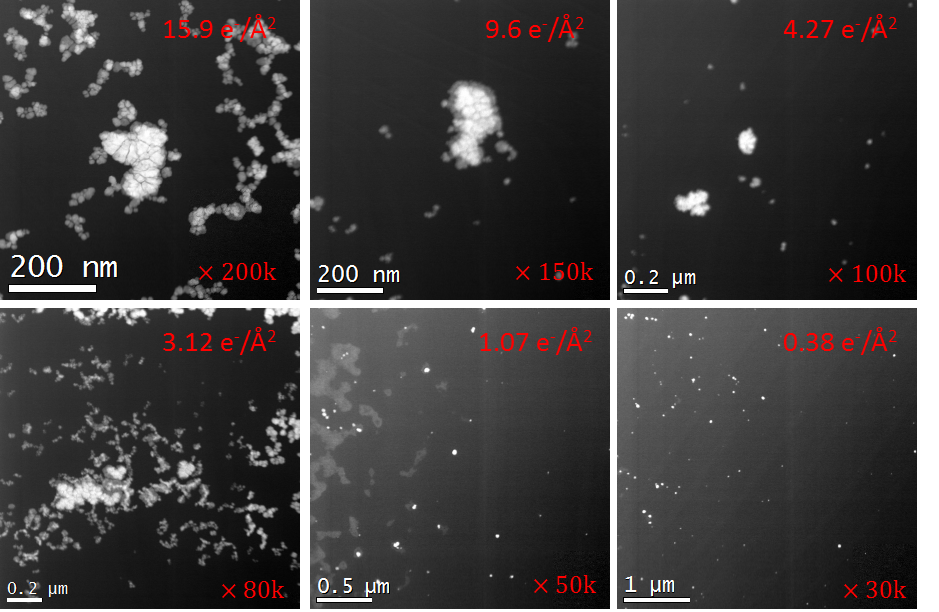


**Figure S9**. The stability of the electrolyte under electron beam irradiation. At the same dwell time (10 μs/pixel) and beam current (50 pA), the electron doses were adjusted by changing magnifications. The bright contrast in the images is from gold nanoparticles which are used as the internal markers. The electrolyte decomposition is identified by the appearance of gas bubbles which show grey contrast in the images.


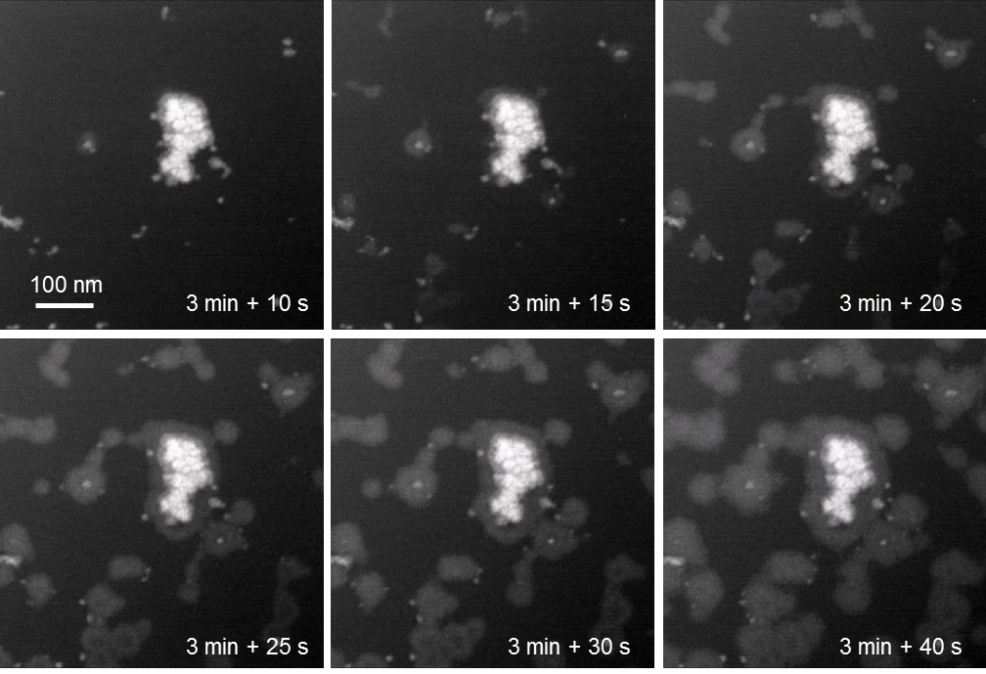


**Figure S10**. At a high magnification of 150k the electrolyte decomposition takes place after 3 min with the formation of bubbles with grey contrast (dwell time: 10 μs/pixel, electron dose: 9.6 e^-^/Å^2^). The bright contrast in the centers of the images is from gold nanoparticles which act as the internal marker.


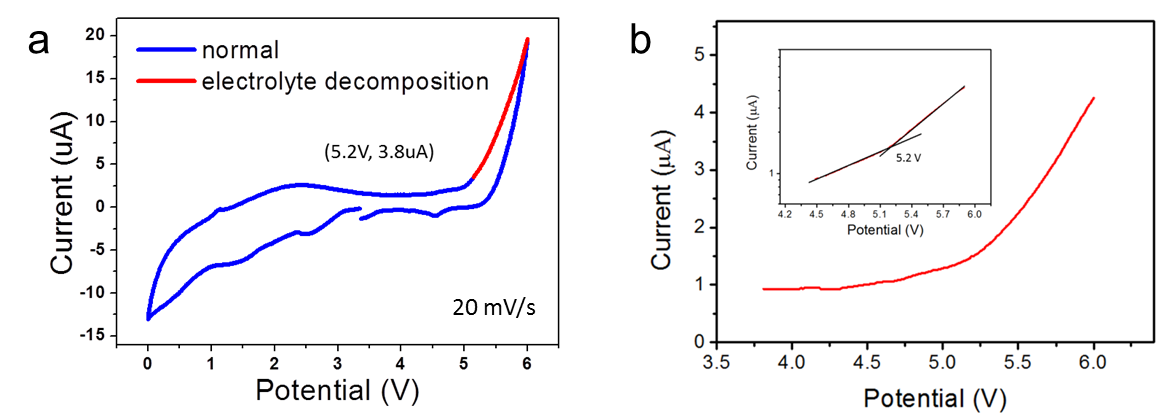


**Figure S11**. (**a**) CV curve collected with the liquid cell using a wide potential range of 0-6V. Electrolyte decomposition occurs at potentials higher than ~5.2 V, which is higher than the maximum voltage (5.0 V) in our operando observations. The sweep rate is 20 mV/s. (**b**) Current-voltage response of the liquid cell during charging. The potential was scanned from the open-circuit voltage to 6 V (without the discharge process) at the rate of 1.0 mV/s. Again, the serious electrolyte decomposition occurs at potentials above 5.2 V.


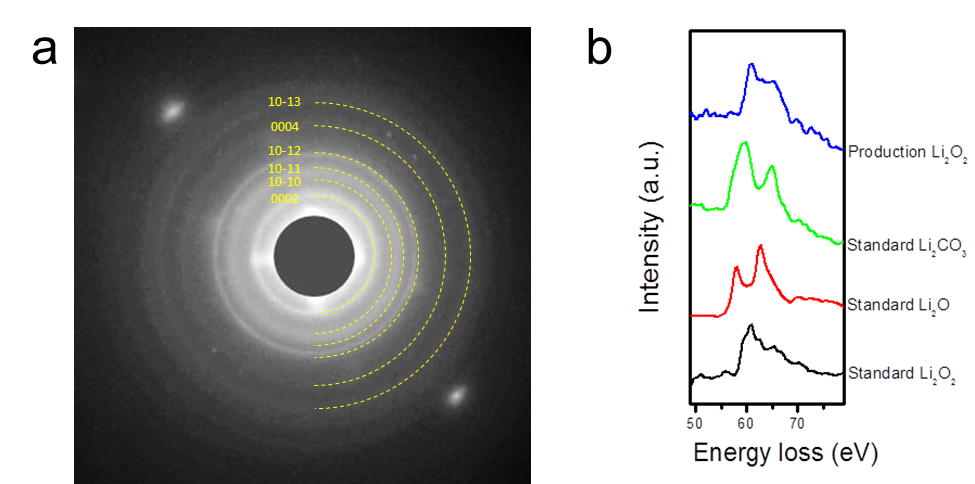


**Figure S12**. The selected area electron diffraction (SAED) and TEM-EELS analysis of reaction products under low acceleration voltage of 120kV. The reaction products were loaded onto the vacuum sample transferring holder inside the glovebox, to avoid the CO_2_/H_2_O/O_2_ attack from atmospheric environment.


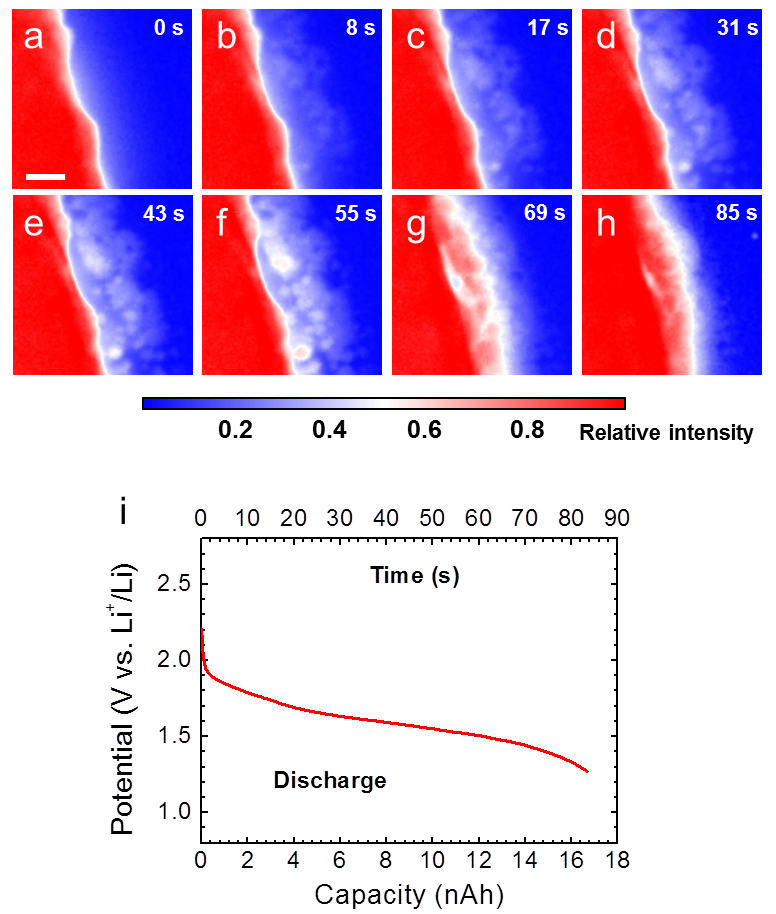


**Figure S13.** In situ electrochemical STEM observations of the fully discharged Au cathode. The passivation of the cathode by the formation of a solid Li_2_O_2_ film leads to the drop of discharge overpotential and the failure of the Li-O_2_ micro-battery. (**a** to **f**) Formation of porous Li_2_O_2_ layers by the accumulation of Li_2_O_2_ nanoparticles. (**g** and **h**) Formation of a dense Li_2_O_2_ film by continuous agglomeration of Li_2_O_2_ nanoparticles. The passivation results in the obvious drop of discharge potential at about 69 s. (scale bar: 100 nm, magnification: 50 k, dwell time: 5 μs/pixel, electron dose: 0.53 e^−^/Å^2^)

**Figure S14**. ANSYS Simulation of the electric field distribution around the Au electrode during the galvanostatic charge. (**a**) The hot spot was localized at the top of the Au working electrode. The electrolyte is DMSO. The landscape shows that during electrochemical cycling the in situ EC STEM exhibits a nonuniform electric field distribution along the Au working electrode. The enlarged image extracted from the red frame. (**b**) The red curve corresponds to the relationship between STEM intensity and position; the green curve corresponds to the relationship between electric filed intensity and position. The enhanced bright contrast of the diffuse layer around the electrode may come from the polarized or charged organic molecules of the electrolyte in the electric field. These charged or polarized molecules with increased ordering (alignment) or density (decrease of inter-molecule distance) could give rise to increased electron scattering for brighter HAADF-STEM contrast.


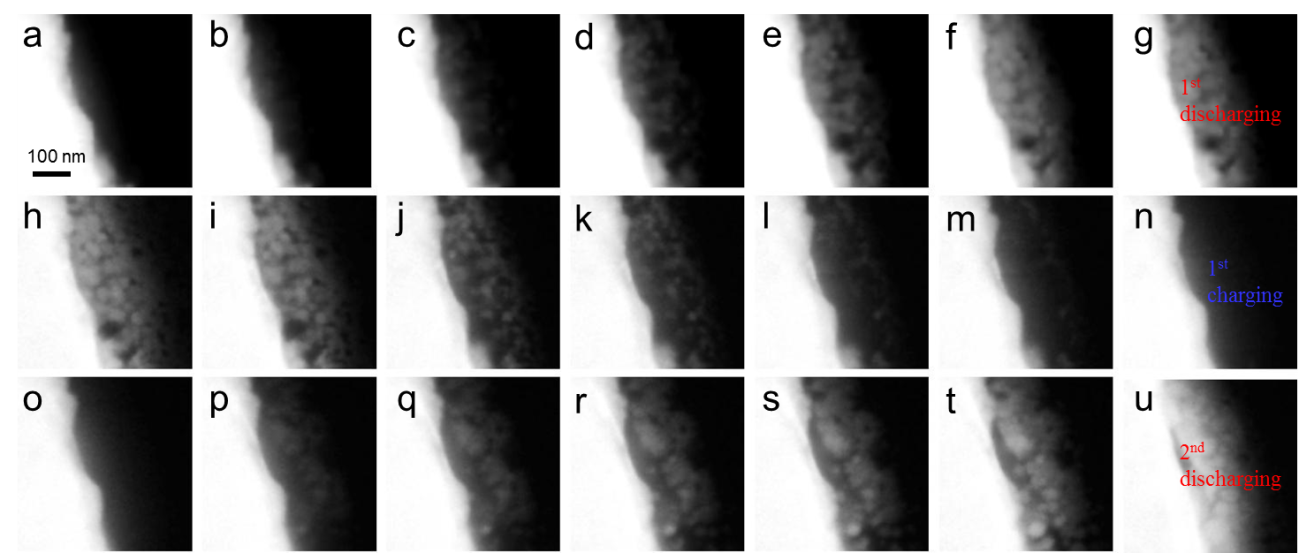


**Figure S15**. Compared with the false colored images in **Figure 2**, the original grey HAADF-STEM images with enhanced contrast are shown here.

**Figure S16**. Schematic illustration of the morphological evolution of Li_2_O_2_ productions under the real time discharge/charge in a micro Li-O_2_ battery.


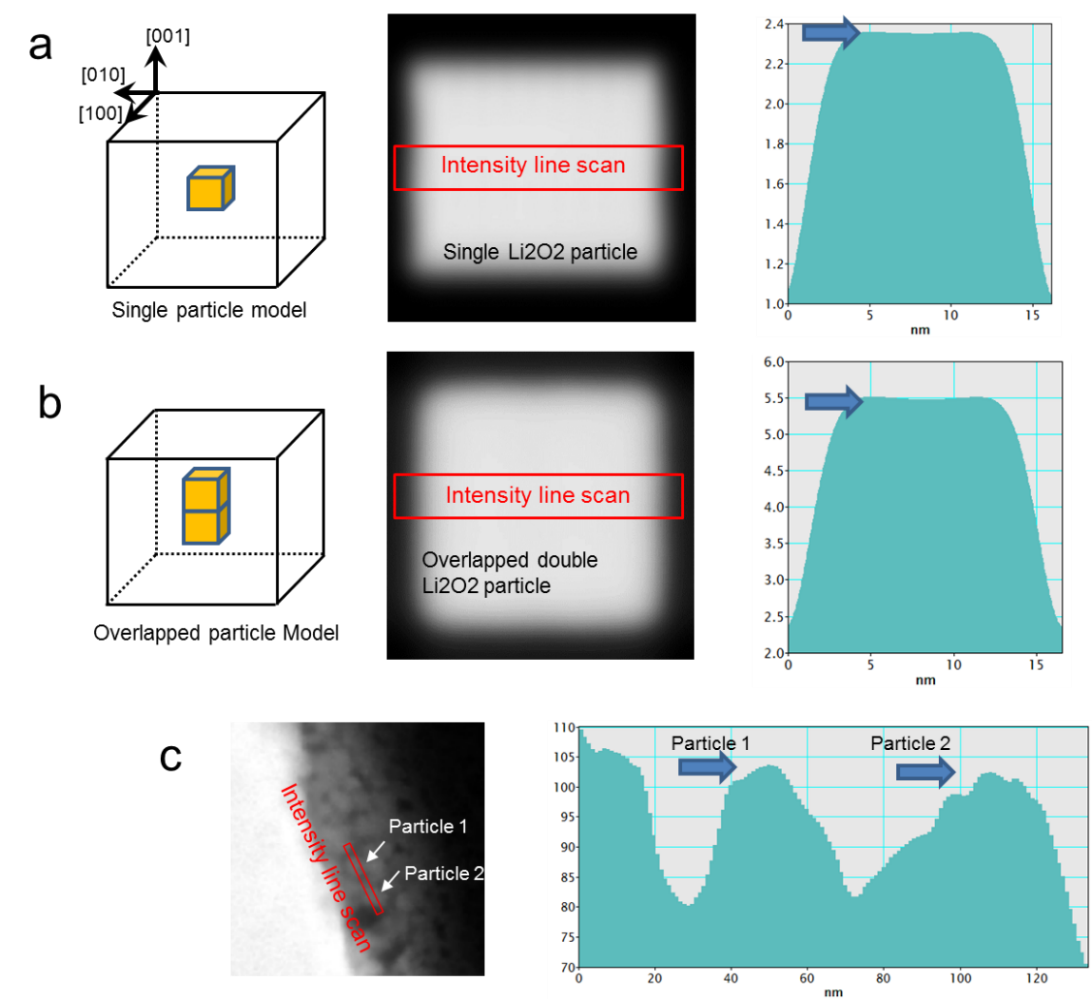


**Figure S17**. HADDF STEM simulations of the contrast in HAADF images of 10 nm Li_2_O_2_ nanoparticles. Li_2_O_2_ shows enhanced contrast (~2.3 times) which only happens when two Li2O2 nanoparticles are perfectly overlapped.

**Table S3**. HAADF STEM simulation parameters

| Acceleration voltage | 200 kV |
| --- | --- |
| Probe size (full-width at half-maximum) | 60 Å |
| Defocus C1 | 0 Å |
| Spherical aberration constant C3 | 0.002 mm |
| Higher order aberration coefficients C5 | 15 mm |
| Objective aperture semi-angle | 30 mrad |
| ADF connection angle | 100-267 mrad |
| Full width at half maximum height of the source image | 1.1 Å |
| Point resolution in HAADF STEM image | 80×80 pixel |
